# Femtosecond Laser Direct Writing of Conductive Carbon Layers on Flexible Kapton Polyimide: A Parametric Study of Process, Microstructure, and Electrical Properties

**DOI:** 10.3390/molecules31203748

**Published:** 2026-10-19

**Authors:** Ahmad S. Barham, Mohammad I. Alkhatab, Wissam Helal, Flavie Braud, Jean-Pierre Vilcot, Basma Elzein

**Affiliations:** 1 Department of Chemistry, School of Science, The University of Jordan, Amman 11942, Jordan; wissam.helal@ju.edu.jo; 2 ADNOC Technical Academy, Al Shamkhah, Abu Dhabi P.O. Box 898/13, United Arab Emirates; malkhatab@adnoc.ae; 3 Univ. Lille, CNRS, Univ. Polytechnique Hauts-de-France, UMR 8520-IEMN-Institut d’Electronique de Microélectronique et de Nanotechnologie, F-59000 Lille, France; flavie.braud@univ-lille.fr (F.B.);; 4 Electrical Engineering Department, College of Engineering, University of Business and Technology, Jeddah 21451, Saudi Arabia; 5 Sustainable Development Department, Global Council for Tolerance and Peace, 1605 Valletta, Malta

**Keywords:** laser-induced carbon conversion, flexible polyimide (Kapton), surface modification, sheet resistance, direct laser writing, flexible electrodes

## Abstract

Femtosecond laser direct writing on Kapton polyimide was investigated using ultraviolet (343 nm) and near-infrared (1030 nm) irradiation. Sixteen conditions were screened optically and electrically. Scanning electron microscopy (SEM) micrographs were obtained for four infrared conditions and one ultraviolet condition; van der Pauw measurements were performed on the ultraviolet- and infrared-processed samples, and X-ray diffraction (XRD) was performed on the infrared sample with the lowest reported sheet resistance (IR-O1). None of the twelve ultraviolet conditions produced a measurable electrical response, whereas all four infrared conditions yielded measurable sheet resistances. The lowest reported sheet resistance, 65.1 Ω/□, was obtained at a repetition rate of 200 kHz, a nominal power setting of 5%, and a scan speed of 20 mm s^−1^. XRD analysis of IR-O1 revealed an additional broad reflection in the graphite (002) region, supporting graphitic stacking in that sample. Bond scission and aromatization of polyimide, as described in the literature, provide a possible molecular pathway to graphitic stacking, although this pathway was not directly investigated. Quantitative SEM analysis did not resolve differences in the apparent open-porosity area fraction across the infrared conditions, despite variations in sheet resistance; the present data do not establish the mechanism responsible for these variations. Because the ultraviolet and infrared conditions differed in repetition rate and nominal power setting, wavelength cannot be isolated as the cause of their different electrical outcomes. These findings highlight the importance of combining electrical and microstructural characterization when assessing femtosecond-laser-modified Kapton.

## 1. Introduction

The growing global energy demand and the need to mitigate greenhouse gas emissions have driven the development of high-efficiency, low-cost photovoltaic systems. In addition to traditional crystalline silicon-based solar cells, various types of third-generation solar cells, such as dye-sensitized, organic, perovskite, and quantum-dot solar cells, are emerging as potential platforms for lightweight, solution-processable and even flexible photovoltaic devices [1,2,3,4,5]. Within such systems, the selection of electrode materials is of utmost importance, as it has a decisive influence on the series resistance, the charge collection efficiency, optical losses, mechanical durability and the long-term stability of the device [2,3,6].

Transparent conducting oxides (TCOs), represented by indium tin oxide (ITO) and fluorine-doped tin oxide (FTO), remain widely used in photovoltaic architectures due to their excellent conductivity and optical transparency [6,7]. Nevertheless, these materials have several critical drawbacks: brittleness, sensitivity to cracking under bending forces, dependence on vacuum-based deposition methods, and incompatibility with roll-to-roll processing on polymer substrates [2,8]. They are also dependent upon rare elements, which impairs cost-effectiveness and supply-chain sustainability [2,6,7,8]. This has led to the search for alternative electrode materials with high electrical conductivity and the other desired characteristics of flexibility, chemical stability and scalability for low-cost processing [2,6,7,8].

Graphene and other carbon-based nanostructures have received significant attention due to their high electrical conductivity, flexibility, stability and robustness [1,6,9]. These properties make graphene a promising material for lightweight flexible photovoltaic devices. Nevertheless, the feasibility of using graphene as an electrode material is related not only to its intrinsic characteristics but also to the possibility of simple, scalable and flexible-substrate-compatible fabrication processes [9,10].

Carbon nanostructures such as graphene and carbon nanotubes have been investigated for a range of functions, such as transparent conductive electrodes, current collectors, buffer layers, hole transport layers and catalytic counter electrodes in dye-sensitized solar cells, organic solar cells and perovskite solar cells [11,12,13]. Graphene-based electrodes have considerable advantages over traditional TCOs such as ITO and FTO in terms of flexibility and compatibility with polymer substrates [14,15]. However, the combination of low electrical resistance, scalable processing and stable integration is still a challenge for photovoltaic applications [16]. For flexible solar cells, it is highly desirable to deposit conductive graphitic layers directly onto polymer substrates without the need for complicated transfer processes [17,18].

Conventional graphene-fabrication routes include solution processing, chemical vapor deposition and epitaxial growth. Although these methods can provide high-quality conductive carbon materials, their application on flexible polymer substrates may require high-temperature processing, transfer, cleaning or subsequent patterning steps, which can increase fabrication complexity and introduce defects or contamination [18,19,20,21,22,23,24,25,26,27,28]. Direct laser conversion of a carbon-rich polymer offers an alternative route in which the conductive carbon layer can be generated and patterned directly on the flexible substrate in a single processing step.

Alternatives to transferred and solution-processed graphene, such as direct-writing techniques, have been gaining attention [29]. Laser-induced conductive carbon (also referred to in the literature as laser-induced graphene (LIG)) has been particularly promising. LIG formation involves the local conversion of a carbon-rich precursor, typically a polymer, into a porous network of graphitic carbon through photothermal and/or photochemical processes whose relative contributions depend on the irradiation conditions. This process can be performed in a single step at room temperature and can be directly patterned using computer-controlled laser scanning, as first demonstrated for laser-induced porous graphene on commercial polymers [30] and subsequently extended to patterned carbon structures on flexible substrates [31,32,33]. Polyimide films such as Kapton are ideal candidates for LIG processing due to their high carbon content, thermal stability, flexibility and cost-effectiveness. When exposed to appropriate laser fluence and wavelength conditions, the Kapton surface can be transformed into porous, electrically conductive carbon structures with varying degrees of graphitic character. The layers can be electrically conductive, highly adherent to the substrate, and inherently patterned along the laser scan direction [30,32,33].

At the molecular level, this transformation has been associated with laser-driven decomposition of the polyimide repeat unit: laser irradiation initiates photothermal and/or photochemical scission of the C–N and C=O bonds of the imide ring, ring opening, and decarbonylation/decarboxylation, releasing CO, CO_2_, and N- and H-containing volatiles; the carbon-enriched residue subsequently aromatizes and condenses into stacked graphitic domains [29,30,32]. The macroscopic outcome, whether a percolating porous graphitic network forms or the process crosses into ablation and delamination, is therefore controlled by the local energy dose delivered by the laser parameters. In the present work, this reaction sequence is used as literature context: the XRD evidence in Section 2.5 is consistent with its graphitic end state, whereas the intermediate bond-cleavage, volatile-release and aromatization steps were not measured.

To date, the literature has demonstrated that laser-induced carbon structures, often referred to as LIG when accompanied by spectroscopic data, can be applied to a broad range of devices. Reviews summarize their use in flexible electronics and sensing [29,34], while individual studies have demonstrated applications including laser-written functional devices and patterned conductive structures [35,36,37,38]. Recent reviews emphasize the advantages of LIG as a fast, maskless and potentially scalable route for generating functional carbon structures [29,34], while experimental studies have demonstrated direct patterning of such structures on polymer substrates [30,35]. LIG is particularly attractive for flexible-electrode applications because it can be formed directly on polymer substrates [29,30] and patterned without additional lithographic processing [35,37].

Ultrashort-pulse conversion of polyimide has previously been demonstrated, including studies relating processing conditions to electrical performance [37]. More broadly, wavelength-dependent studies of polyimide have demonstrated conductive laser-induced carbon formation from the ultraviolet to the infrared, with the productive processing conditions varying substantially with wavelength and irradiation parameters [37]. Reports that describe productive and non-productive femtosecond-processing conditions on Kapton together, as in [39], are useful in this respect. In particular, identifying which recorded processing conditions produce a continuous conductive network, rather than surface modification or ablation, is important for translating femtosecond direct writing toward flexible-electrode applications.

The present study therefore examines a parametric femtosecond-laser processing matrix on Kapton using ultraviolet (UV; 343 nm) and near-infrared (IR; 1030 nm) irradiation. Electrical screening is combined with optical and electron-microscopy observations, calibrated analysis of the apparent open-porosity area fraction in the conductive infrared series, and structural characterization of the infrared-processed sample with the lowest reported sheet resistance. The objective is not to establish an intrinsic ranking of the two wavelengths, because the UV and IR series were acquired over different repetition-rate and nominal-power ranges, but to identify the productive and non-productive regions of the explored parameter space represented in the available dataset and to relate the observed electrical response to the accessible microstructural evidence. Femtosecond conversion of polyimide into conductive carbon has already been reported [39,40,41]. The contribution of this work is therefore an integrated process–morphology–electrical assessment of a femtosecond Kapton processing matrix, showing that, under the tested conditions, visible surface modification and localized pore formation did not necessarily correspond to a measurable electrical response, and that the measured apparent open-porosity area fraction alone did not explain the observed sheet-resistance differences among the investigated infrared conditions. These findings are interpreted within the limitations of the available experimental dataset.

## 2. Results and Discussion

### 2.1. Electrical Screening Overview

For the sixteen conditions listed in Table 1, Table 2 summarizes the electrical outcomes. None of the twelve UV conditions produced a measurable electrical response under the applied van der Pauw procedure, whereas all four IR-O conditions yielded measurable sheet resistances, with one reported value per condition ranging from 65.1 to 95.7 Ω/□ (Section 3.5). These electrical results provide a basis for the microstructural discussion that follows: the UV series identifies a non-productive region of the explored parameter space in terms of measurable electrical response, whereas the IR-O series provides the dataset for evaluating relationships among processing conditions, surface morphology, and electrical response.

### 2.2. UV-L Series: Low-Dose UV Femtosecond Screening

**Figure 1:**
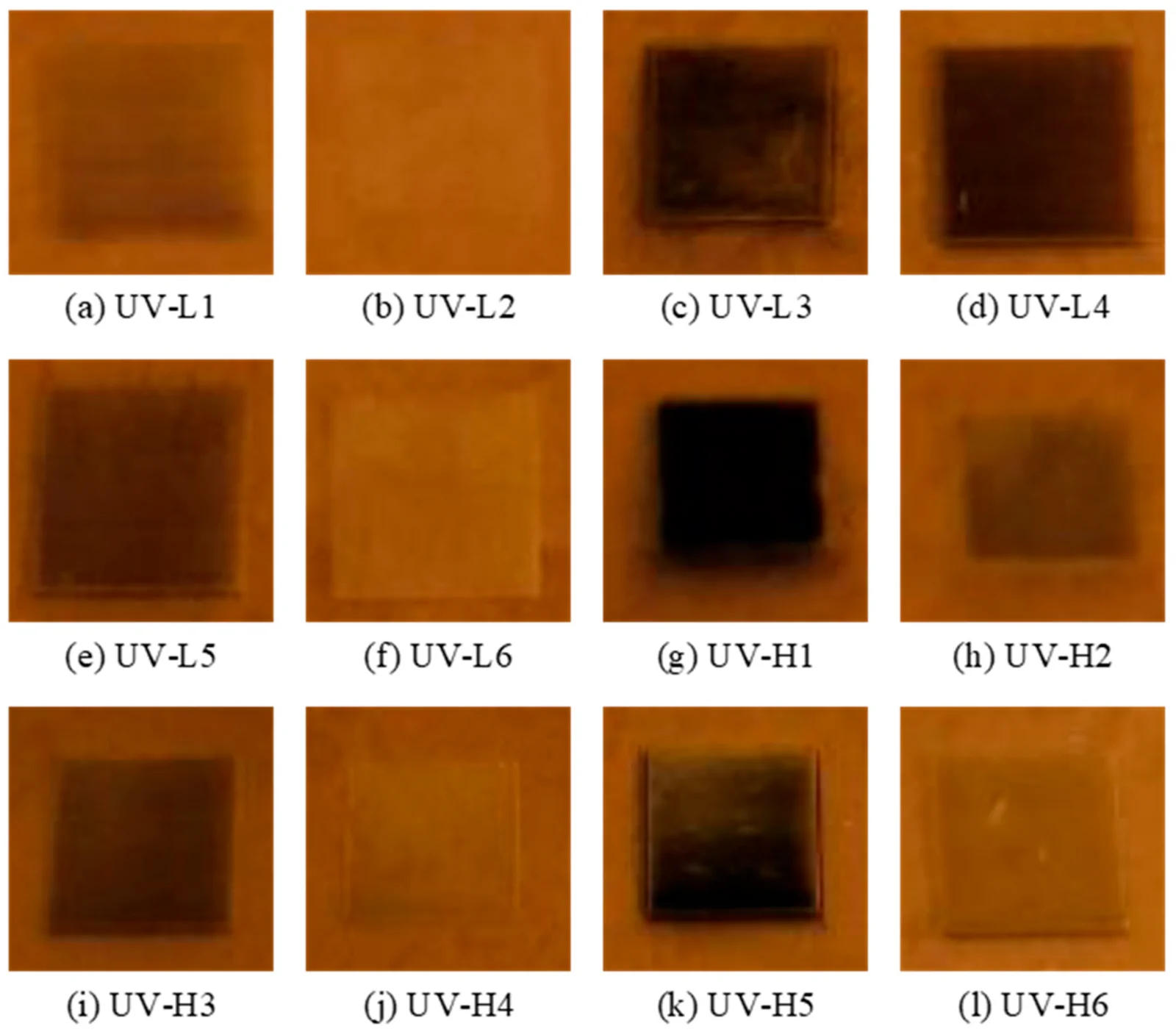
Photographic arrays of Kapton films after femtosecond ultraviolet laser processing under (**a**–**f**) low-dose UV conditions (UV-L series) and (**g**–**l**) higher-dose UV conditions (UV-H series). Panels: (**a**) UV-L1, (**b**) UV-L2, (**c**) UV-L3, (**d**) UV-L4, (**e**) UV-L5, (**f**) UV-L6, (**g**) UV-H1, (**h**) UV-H2, (**i**) UV-H3, (**j**) UV-H4, (**k**) UV-H5, and (**l**) UV-H6. The photographs illustrate qualitative differences in macroscopic darkening and surface appearance after laser treatment. Because image acquisition was not standardized for reflectance or illumination, these photographs are used only for qualitative visual comparison.

The UV-L series was designed as an initial screening study to determine whether low-dose UV femtosecond irradiation could initiate porous conductive surface modification on Kapton. Optical images of the UV-L series are presented in Figure 1. Visible darkening relative to the untreated substrate was observed for UV-L1, UV-L3, UV-L4, and UV-L5, whereas UV-L2 and UV-L6 showed little or no visible darkening in Figure 1. Such darkening is consistent with laser-induced surface modification and the onset of carbonization in polyimide [30,32,36,37], but does not by itself demonstrate the formation of a continuous conductive carbon network.

However, the modified regions remained relatively smooth and lacked the matte, expanded, or strongly textured appearance typically associated with a well-developed laser-converted carbon layer. Thus, although the optical darkening is consistent with surface modification, the extent of structural evolution remained limited.

**Figure 2:**
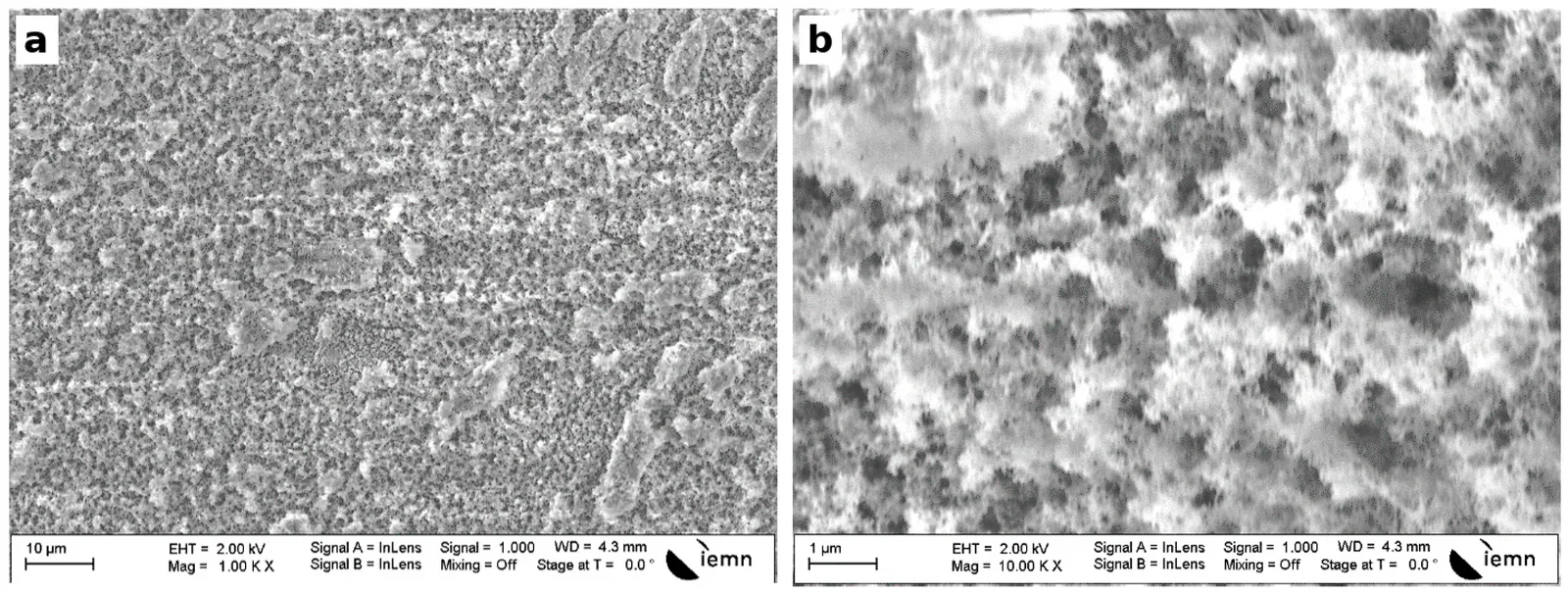
SEM micrographs of UV-L4 showing incomplete surface coverage, fissure-like defects and discontinuous porous regions: (**a**) 1.00 k×, original instrument scale bar = 10 µm, EHT = 2.00 kV, WD = 4.3 mm; (**b**) 10.00 k×, original instrument scale bar = 1 µm, EHT = 2.00 kV, WD = 4.3 mm. EHT = electron high tension; WD = working distance. Original instrument databars are preserved in both panels.

SEM was used as a screening tool across the laser-written arrays (Section 3.4). Within the UV parameter space, UV-L4 (50 kHz, 15% nominal power setting, 20 mm s^−1^) was the only condition in which a developed porous surface morphology was observed under SEM inspection; the remaining UV conditions showed no clearly developed structural conversion, and micrographs are therefore available only for UV-L4 [42]. Figure 2 shows this condition: partial surface coverage, fissure-like cracks, and porous ligaments. The micrographs are interpreted qualitatively. Their fragmented morphology is consistent with incomplete surface conversion and with the absence of a measurable electrical response, but no quantitative coverage or connectivity metric is reported here. This matches the outcome recorded for the entire UV-L series (Table 2). The UV-L series therefore represents a partially converted UV regime: surface darkening was observed in most conditions of the series, and some pore formation was observed in UV-L4.

### 2.3. UV-H Series: Higher-Dose UV Femtosecond Screening

The UV-H series was designed to probe a higher nominal energy-input region, to explore whether the surface modification could be pushed into an open porous structure with better conductivity. This series is shown in Figure 1. Visible darkening was observed for UV-H1, UV-H2, UV-H3 and UV-H5, whereas UV-H4 and UV-H6 showed little visible darkening relative to the surrounding Kapton in Figure 1. However, the modified regions still appeared optically opaque and relatively flat, without a pronounced matte or frothy appearance. The optical appearance is therefore more indicative of surface modification and/or limited ablation than of the formation of an open, conductive carbon network.

UV-H1 and UV-H2 were recorded at the lowest repetition rate and the highest nominal power setting in the ultraviolet matrix. Because the delivered dose cannot be reconstructed (Section 3.2), these conditions are reported by their recorded settings only. Their modified regions showed no evidence of topographic expansion. Among the mid- to high-repetition-rate treatments, UV-H3 and UV-H5 exhibited more pronounced and relatively uniform darkening. UV-H4 and UV-H6 showed the least apparent darkening in Figure 1. Because image acquisition was not standardized (Section 3.3), these visual observations are qualitative and are not used to quantify the extent of material conversion. The UV-H array was likewise screened, and no condition in it produced a developed porous surface morphology or a measurable electrical response [42]; micrographs are consequently not available for this series, and its interpretation is limited to the optical response, the documented negative screening outcome, and the process trend. In the UV parameter space considered, the UV-H conditions, recorded at higher nominal power settings than the UV-L conditions, exhibited more uniform darkening in some cases (UV-H3 and UV-H5), but none produced a measurable electrical response or evidence of developed network formation.

### 2.4. IR-O Series: IR Optimization at Constant Repetition Rate and Nominal Power Setting

**Figure 3:**
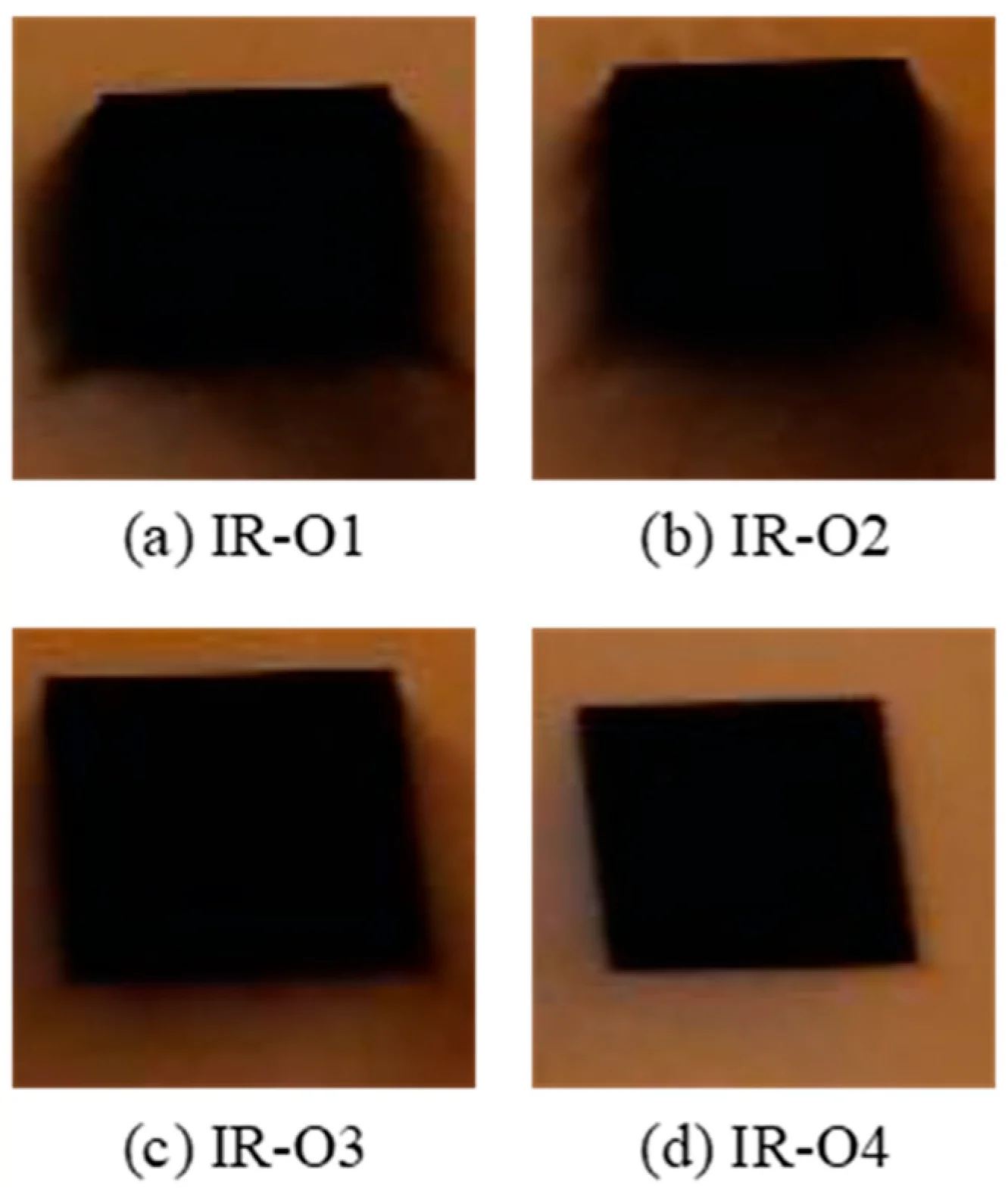
Photograph of the Kapton sample array processed under the IR-O optimization conditions: (**a**) IR-O1, (**b**) IR-O2, (**c**) IR-O3, and (**d**) IR-O4. Because image acquisition was not standardized for reflectance or illumination, these photographs are used only for qualitative visual comparison.

In this set, repetition rate and nominal power setting were fixed at 200 kHz and 5%, while scan speed was varied between 20 and 50 mm s^−1^ to study the influence of scan speed on the surface morphology and sheet resistance. In contrast to the UV sets, SEM micrographs and quantitative sheet-resistance values, in addition to optical images (Figure 3), are available for all four IR-O conditions, allowing morphology and sheet resistance to be examined together.

Optically, the IR-O samples showed differences in darkness, gloss, and apparent surface texture across the scan-speed series. IR-O1 appeared darker and grayer, whereas IR-O3 and IR-O4 appeared smoother and more semi-glossy. Because the optical images were not acquired under standardized reflectance or illumination conditions, these observations are used only as qualitative indicators of surface appearance and are not interpreted as quantitative evidence of differences in open porosity.

**Figure 4:**
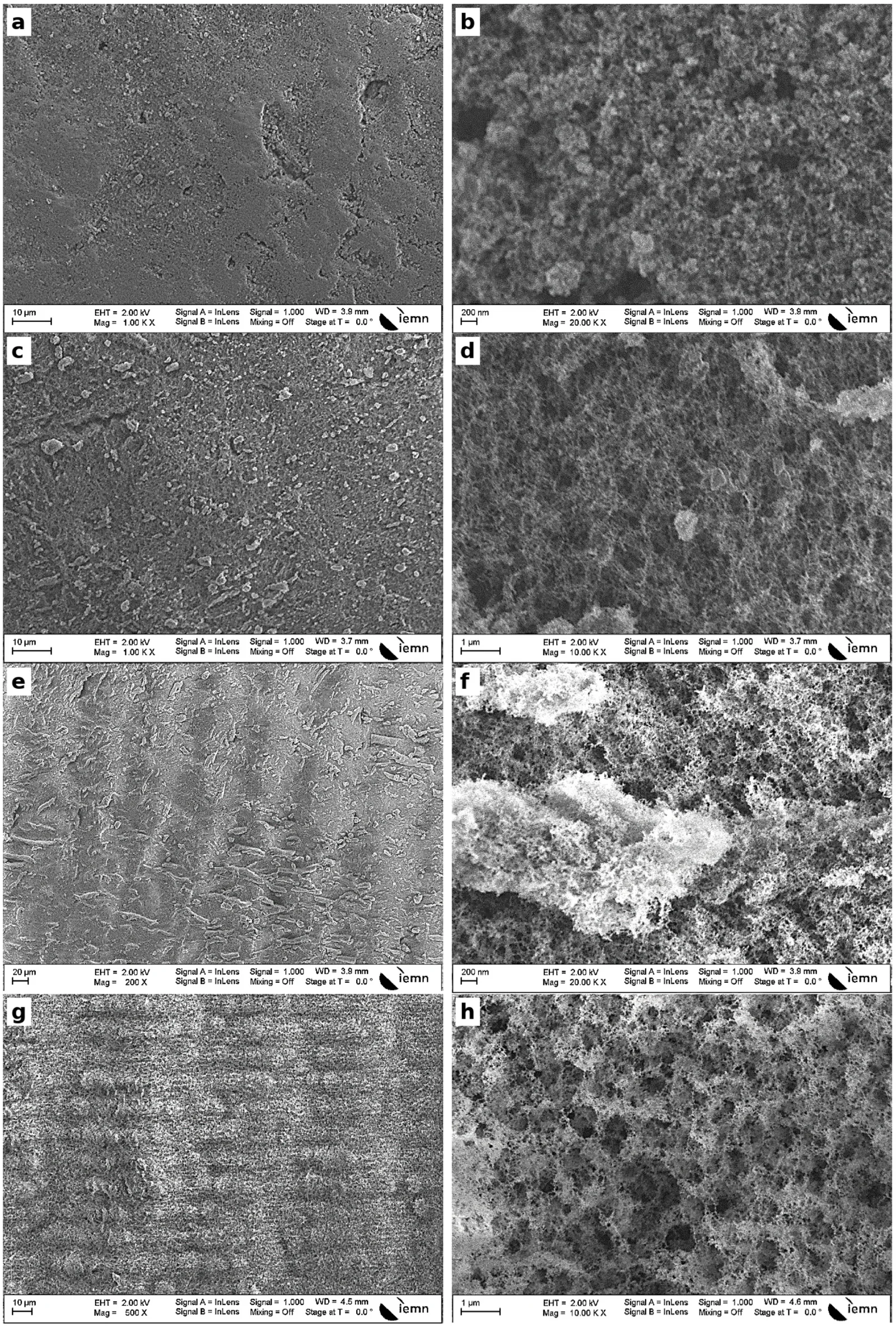
SEM micrographs of the IR optimization (IR-O) series at a fixed repetition rate of 200 kHz and a 5% nominal power setting with increasing scan speed: (**a**,**b**) IR-O1; (**c**,**d**) IR-O2; (**e**,**f**) IR-O3; (**g**,**h**) IR-O4. Each condition is shown at a broader and a finer length scale. The magnifications were not standardized across conditions: (**a**) 1.00 k×, (**b**) 20.00 k×, (**c**) 1.00 k×, (**d**) 10.00 k×, (**e**) 200×, (**f**) 20.00 k×, (**g**) 500×, and (**h**) 10.00 k×. The panels are therefore presented for qualitative morphological illustration and are not quantitatively compared with one another. All images were acquired at 2.00 kV with an in-lens detector, at working distances of 3.9 mm (**a**,**b**), 3.7 mm (**c**,**d**), 3.9 mm (**e**,**f**), 4.5 mm (**g**), and 4.6 mm (**h**). Each panel keeps its original instrument scale bar and databar; no scale bar has been added or redrawn. The quantitative comparison in Table 3 is made separately, on calibrated source images brought to a common field of view and pixel sampling (Section 3.6).

Qualitatively, IR-O1 appeared to exhibit a relatively open and interconnected porous morphology (Figure 4). However, because the SEM micrographs were acquired at different magnifications, these visual impressions should be treated cautiously, and no relationship between this morphology and the sheet resistance is inferred from them.

Table 3 reports the apparent open-porosity area fraction of the IR-O series, obtained from micrographs resampled to a common field of view and pixel sampling (Section 3.6). Pore-size and ligament-width descriptors are not reported for the reasons given in Section 3.6. The IR-O3 field contains a pronounced bright agglomerated region and consequently shows the largest within-field variability; the feature was retained in the analyzed field, and no image processing parameters were altered on account of it. The mean apparent open-porosity area fraction ranged from 53.4% to 59.9%, corresponding to a between-condition range of 6.5 percentage points. The ± values in Table 3 are standard deviations over six spatial sub-fields of a single field of view for each condition; they describe within-field spatial variability only and are neither experimental uncertainties nor measures of reproducibility between independently processed samples (Section 3.6). The largest within-field standard deviation was 4.8 percentage points, whereas the sensitivity of the apparent open-porosity area fraction to the predefined threshold perturbation ranged from 7.3 to 17.8 percentage points (Table 3). This sensitivity exceeded the 6.5-percentage-point range of the reported mean values across the four IR-O conditions in every case. Accordingly, the observed between-condition variation was not considered resolved within the sensitivity of the available image analysis. Because the comparison rests on one analyzed field per condition (Section 3.6), the dataset does not support an equivalence test between conditions, and these results do not establish that the pore architecture is invariant. The 47% increase in reported sheet resistance from IR-O1 to IR-O3 (one reported value per condition, without a quantified measurement uncertainty; Section 3.5) is consequently not accounted for by the measured apparent open-porosity area fraction, whose mean values do not vary monotonically with scan speed across the series. The present data cannot establish the mechanism responsible for this resistance increase. Layer continuity across adjacent scan lines (6.7 µm hatch spacing) and converted layer depth remain untested and require larger-area imaging and cross-sectional profiling (Section 2.7).

### 2.5. XRD Characterization of the Lowest-Sheet-Resistance Layer (IR-O1)

**Figure 5:**
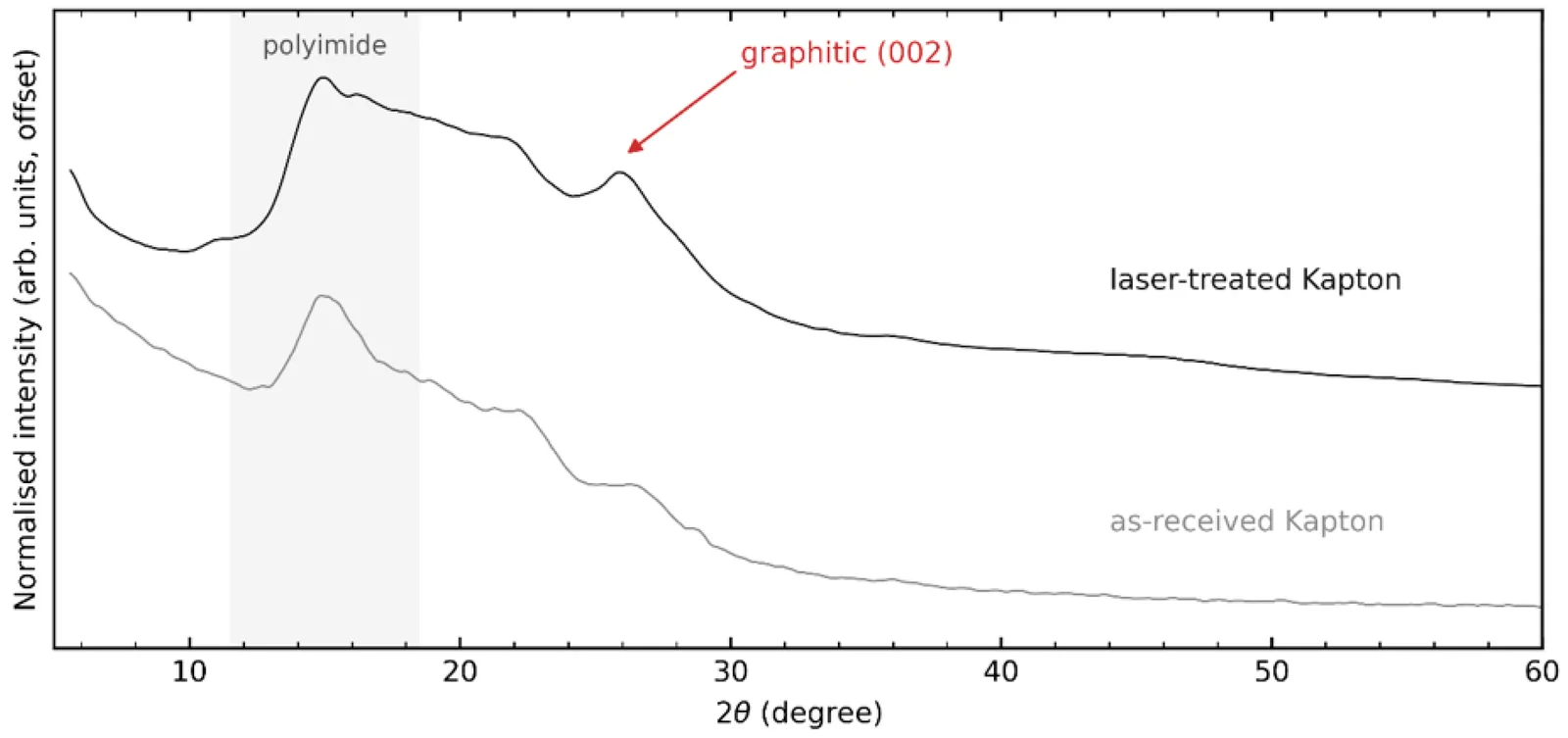
X-ray diffraction patterns of laser-treated Kapton (IR-O1) compared with as-received Kapton. The laser-treated material shows an additional broad reflection in the graphite (002) region near 26° 2θ; no comparable distinct peak is evident in the untreated Kapton pattern. The diffraction patterns are interpreted qualitatively, and no peak-fitting-derived parameters are reported.

The X-ray diffraction measurement acquired on IR-O1, the infrared-processed sample with the lowest reported sheet resistance (65.1 Ω/□), is presented in Figure 5 [42]. The diffractogram is dominated by the broad scattering signature of the Kapton substrate at low angles, with an additional broad reflection in the graphite (002) region, near 26° 2θ, in the laser-treated material. Figure 5 also shows the pattern of an as-received Kapton film recorded using the same θ–2θ measurement geometry; no comparable discrete reflection is evident in the untreated substrate, supporting attribution of the additional feature to the laser-modified layer. The diffraction measurement is averaged over the illuminated area and therefore supports the presence of graphitic stacking within the laser-modified region but does not localize that phase to any particular feature resolved by electron microscopy. At the molecular level, such stacking is the expected end product of the imide-ring scission, volatile release and aromatization sequence reported for laser-converted polyimide [29,30]; the present measurement is consistent with this end state but does not resolve the intermediate steps. This pathway is therefore a literature-based interpretation, drawn from studies of laser-converted polyimide [29,30,32] and from related thermal-pyrolysis and simulation studies [43,44,45], and not an experimental finding of the present study; the findings demonstrated experimentally here are limited to the optical appearance (Figure 1 and Figure 3), the electrical response (Table 2), the surface morphology (Figure 2 and Figure 4; Table 3) and the XRD feature of IR-O1 (Figure 5). SEM is used throughout this work as morphological evidence only: it resolves the surface texture and porosity of the converted layer but does not by itself establish electrical or structural continuity across adjacent scan lines. It also cannot distinguish graphitic carbon from amorphous or ablated carbonaceous material, and no individual feature imaged by SEM is identified here as graphitic based on its appearance. Raman mapping and X-ray photoelectron spectroscopy (XPS) would be needed to localize the graphitic phase relative to the features observed by SEM and to characterize graphitic ordering, defects, and surface chemistry (Section 2.7). On the basis of the XRD evidence, the IR-O1 layer is described in this work as laser-induced graphitic carbon. XRD was performed on this sample only; graphitic stacking is therefore not directly established for IR-O2–IR-O4, which are referred to as conductive laser-converted carbon layers, and no compositional designation is applied to the ultraviolet-processed conditions. The term “graphitic” is therefore used in this work only for IR-O1; where the laser-converted layers are discussed collectively, they are referred to as conductive laser-converted carbon.

### 2.6. Comparative Assessment of UV and IR Processing

The three processing sets show a clear difference in outcome between the recorded UV and IR femtosecond-processing conditions. UV processing (UV-L and UV-H, Figure 1) produced visible darkening in most conditions, suggesting surface modification in those cases, but did not produce clear evidence of an open porous conductive network. The only ultraviolet condition for which micrographs are available, UV-L4 (Figure 2), exhibited localized porosity, incomplete surface conversion, fissure-like cracks, and discontinuous porous ligaments, without an evidently continuous, interconnected network. These morphological observations are consistent with the absence of a measurable electrical response under the applied van der Pauw procedure, but do not independently establish the absence of electrical transport. The remaining ultraviolet conditions were assessed only at the screening level (Section 2.2 and Section 2.3). These findings indicate that none of the investigated UV processing conditions yielded a measurable electrical response. However, the available dataset does not establish the boundaries of a processing parameter range capable of producing a conductive carbon layer.

Why the explored ultraviolet conditions did not yield a conductive layer cannot be resolved from this dataset. The ultraviolet and infrared conditions were not recorded over matched parameter ranges: the ultraviolet conditions were run at repetition rates of 4–50 kHz and nominal power settings of 15–50%, whereas the infrared conditions were run at 200 kHz and a nominal power setting of 5% (Table 1). No condition is common to the two sets, no calibration relating attenuator settings below 100% to the average power delivered to the sample is available, the focused spot diameter at the sample was not measured, and no pulse duration was measured for the 343 nm output (Section 3.2). The two series therefore cannot be placed on a common fluence or dose axis, and wavelength cannot be isolated as the sole causal variable distinguishing the two outcomes.

The studies discussed below show that productive conditions differ between wavelengths, so such a contrast is better read as a question of whether each tested condition series coincides with productive conditions for that wavelength than as a property of the wavelength itself. Ultraviolet irradiation of polyimide can form laser-induced graphene: high-quality material has been obtained at 355 nm after process optimization [46], and ultraviolet-formed laser-induced graphene has been reported with an approximately fourfold reduction in penetration depth relative to the conventional infrared source [36]. The productive conditions, however, depend on irradiation parameters and wavelength: conductive laser-induced graphene has been produced on polyimide at 355, 532 and 1064 nm, with the productive irradiation conditions differing between wavelengths [37], and in one 355 nm polyimide study high-quality material was obtained only over a restricted fluence interval, while tighter focusing increased the tendency toward ablation [47]. In a separate femtosecond 1030 nm study of polyimide, carbonization was reported at low power and low scan speed, whereas clean ablation occurred under more aggressive processing conditions [39].

An interpretation consistent with the present observations is therefore that the recorded ultraviolet conditions did not coincide with productive conditions for that wavelength, giving surface modification and, in UV-L4, a discontinuous morphology (Figure 2), without the continuous network expected to support a measurable electrical response, whereas the investigated infrared conditions yielded measurable sheet resistances. Wavelength-dependent optical absorption and energy-deposition pathways reported in the literature [37,39,48,49,50] may contribute to this contrast by altering the balance among surface modification, carbonization, gas evolution, network formation, ablation and delamination; this was not tested in the present work.

All laser processing in the present study was performed in ambient air, so oxygen was available during irradiation and may have participated in surface oxidation and in the competition between carbonization and material removal. An influence of the processing atmosphere has been reported in other studies [32,37,51]. However, the present dataset does not include atmosphere-controlled experiments or surface-chemical analysis, and the specific contribution of oxygen therefore cannot be quantified. Determining this contribution would require matched processing under controlled atmospheres together with chemical characterization such as XPS.

The absorption literature cited in Section 3.1 provides context only. For the reasons given above, the parameter-space explanation proposed in this section remains an interpretation consistent with the observations rather than a measured mechanism. Placing both wavelengths on a common fluence axis, using measured spot size and delivered average power, would require a new sample matrix (Section 2.7).

The original screening matrix extended beyond the sixteen conditions reported in Table 1 and included more aggressive IR conditions, under which subtractive processes dominated. These conditions produced extensive peeling and delamination from the substrate, leaving residual micro-islands and a sponge-like aggregated residue; at higher nominal power (attenuator) settings, substrate damage became severe [42]. The productive IR-O conditions reported here therefore lie below more aggressive conditions that produced ablation and delamination; the position of this upper boundary was not determined (Section 2.7). The lower boundary of the productive IR conditions was not located within the IR-O set, since all four scan speeds examined remained conductive. This carbonization-versus-ablation competition is consistent with the dose dependence reported for ultrashort-pulse and CO_2_-laser processing of polyimide [33,37]. Direct mass-loss and depth-profiling measurements were not part of the original matrix (Section 2.7). The flexibility of an infrared-processed layer was assessed qualitatively during the original matrix: the film remained intact under bending, with cracking onset observed at approximately 60–70° of manual flexure [42]; standardized cyclic bending tests remain to be performed.

Within the IR-O series, measurable sheet resistance was obtained across the full range of scan speeds examined, with the reported values increasing from 65.1 Ω/□ for IR-O1 to 95.7 Ω/□ for IR-O3, while IR-O4 yielded 93.3 Ω/□. Because the individual resistance readings underlying the reported values are unavailable and no measurement uncertainty can be assigned (Section 3.5), the statistical distinguishability of the IR-O3 and IR-O4 values, which differ by less than 3%, cannot be assessed using the available measurement information. The larger differences across the series are likewise discussed as observed differences between reported values rather than as statistically established effects. Quantitative image analysis (Section 3.6; Table 3), however, showed that the variation in electrical response was not accompanied by a resolvable difference in apparent open-porosity area fraction, which also did not vary monotonically with scan speed.

The electrical variation observed within the IR-O series therefore cannot be attributed to the measured apparent open-porosity area fraction. Instead, it may arise from structural characteristics at larger length scales that were not resolved by the present SEM analysis, including the continuity of the converted carbon layer across adjacent scan lines (6.7 µm hatch spacing) or variations in the converted-layer depth; the present data cannot establish the mechanism responsible.

Among the four investigated IR-O conditions, IR-O1 exhibited the lowest reported sheet resistance (65.1 Ω/□), and this is the only basis on which it is distinguished in this work. Because each condition is represented by a single sheet-resistance value, without independent replicate samples or an assignable measurement uncertainty (Section 3.5 and Section 2.7), the differences between IR-O1 and the other IR-O conditions are not statistically established, and IR-O1 is not claimed to be an optimal processing condition. Overall, within the conditions investigated here, the infrared settings produced carbon layers with measurable sheet resistance, whereas the ultraviolet settings yielded no measurable electrical response under the applied electrical characterization procedure. This comparison concerns the investigated parameter sets as a whole and does not establish an intrinsic ranking of the two wavelengths.

These sheet resistances can be compared with published values for laser-converted polyimide, with the caveat that laser regime, substrate thickness and measurement geometry differ between studies. Using 10 ps pulses on 127 µm Kapton in air, Fiodorov et al. reported minimum sheet resistances of 18, 13 and 8 Ω/□ at 355, 532 and 1064 nm, respectively, and 5 Ω/□ at 1064 nm under a nitrogen atmosphere [37]. In a separate 10 ps, 1064 nm study on 127 µm Kapton in air, Sartanavičius et al. obtained a minimum of 36.6 Ω/□ and reported values on the order of 100 Ω/□ over part of the productive irradiation-dose window [52]. Outside the ultrashort-pulse regime, CO_2_-laser processing of approximately 127 µm polyimide in air gave a sheet resistance below 35 Ω/□ at the graphitization threshold and a minimum below 15 Ω/□ at higher laser power, with unconverted polyimide exceeding 90 MΩ/□ [30]. The values obtained here, 65.1–95.7 Ω/□, therefore fall within the range reported in the picosecond near-infrared studies of polyimide cited above, while remaining above the optimized values reported in those studies. Three differences limit how closely the numbers can be compared. First, the cited studies used Kapton of approximately 127 µm, against 50 µm here, so the precursor thickness available for conversion is not matched. Second, the present values were obtained in the van der Pauw configuration, which may differ from the probe geometries used in the cited studies, so the measurement geometries are not directly matched. Third, the lowest value reported in the literature cited above was obtained under nitrogen [37], whereas all processing reported here was performed in ambient air. The comparison is accordingly indicative of where the present tested conditions sit, rather than a quantitative ranking. Recent femtosecond studies on polyimide have also reported conductive laser-induced carbon under optimized conditions [40,41]; differences in laser parameters, substrate characteristics and electrical measurement configurations prevent direct quantitative benchmarking against the present results.

The principal advantages of the present femtosecond direct-writing approach are maskless patterning, room-temperature processing, direct conversion of the polymer surface without transfer or wet-chemical deposition, and compatibility with flexible substrates. The present dataset does not include a nanosecond-laser comparison, and therefore no experimental advantage of femtosecond over nanosecond processing is claimed here. The limitations of the approach in its present form include the undetermined extent of the productive parameter range, sensitivity to laser-processing conditions, the unknown delivered fluence in the available dataset, the absence of compositional characterization, and the need to characterize and control layer continuity, thickness and electrical reproducibility. These trade-offs are consistent with broader studies of laser-synthesized conductive carbon from polymer films, in which conductivity and structural quality depend strongly on both precursor properties and laser-processing conditions [53].

### 2.7. Limitations

The scope of the present conclusions is delimited as follows.

(i) Raman mapping and X-ray photoelectron spectroscopy (XPS) were not performed in this study. The XRD feature in the graphite (002) region supports the presence of graphitic stacking averaged over the illuminated area, but not the degree of ordering, the defect density, the sp^2^/sp^3^ balance, the surface chemistry, or the spatial distribution of the graphitic phase within the converted layer. Because electron microscopy cannot discriminate graphitic from amorphous or ablated carbonaceous material on morphological grounds, the association between the imaged porous structures and the diffracting graphitic phase is inferred rather than demonstrated. The IR-O1 layer is accordingly described as laser-induced graphitic carbon rather than laser-induced graphene; because XRD was performed on this sample only, this designation is not directly established for IR-O2–IR-O4. No diffraction measurement was made on any ultraviolet-processed condition, so no compositional assignment is made for the UV series. The chemical composition of the converted layer, including elemental composition, sp^2^/sp^3^ balance and surface chemistry, is therefore not determined in this work.
(ii) SEM micrographs are available for the four IR-O conditions and UV-L4 only; the UV-H series is interpretable only at the screening level, based on optical observations and the absence of a measurable electrical response. The ultraviolet conditions were electrically characterized using the same van der Pauw procedure as the infrared conditions; however, the instrument-specific detection limit cannot be reconstructed from the available records (Section 3.5).
(iii) For each condition analyzed, quantitative morphological data were extracted from one field of view using the highest-magnification micrograph available for that condition; further micrographs are available at other magnifications (Figure 4) but were not used for quantification. The reported dispersion describes within-field spatial variability only (Section 3.6). Experimental reproducibility between independently processed samples cannot be quantified from the available dataset because no replicate-sample datasets are available and the underlying electrical measurement records are no longer available. The reported electrical values therefore represent one sheet-resistance value per IR-O condition, without recoverable individual readings or documented independent sample-level replicates. The apparent open-porosity area fraction is the only quantitative morphological descriptor reported (Section 3.6). The dataset does not establish morphological equivalence between conditions, and no inferential statistics are reported for these descriptors.
(iv) No atomic force microscopy (AFM) dataset suitable for the present parametric analysis is available. Surface topography over areas spanning multiple scan lines, and its relation to the three-dimensional morphology of the porous network, therefore remain uncharacterized for this sample set.
(v) Sheet resistance is reported as measured; no layer thickness was determined for this sample set, and no bulk resistivity or conductivity is claimed. No statistical uncertainty can be estimated for the reported sheet resistances, and the systematic contributions identified in Section 3.5 could not be quantified; no combined uncertainty is therefore assigned. The van der Pauw assumptions of a homogeneous, isotropic layer of uniform thickness are only approximately satisfied by a porous laser-converted layer (Section 3.5). No cross-sectional or depth-resolved measurement of the converted layer was made during the screening matrix; determining the converted depth requires cross-sectional profiling on a new sample set.
(vi) The bending observation is qualitative.
(vii) Mass-loss, depth-profiling, and cross-sectional measurements of the subtractive regimes were not performed.
(viii) The optical configuration of the processing system is documented (Section 3.2), including the models and nominal focal lengths of the F-theta lenses, the 1/e^2^ beam diameters before the lenses (measured after the screening matrix with the same optical configuration) and the 100% reference powers at 200 kHz measured at the sample level. However, the focal position was located empirically from test marks and the focused spot diameter at the sample was not measured, the beam-expander magnification was not documented, no calibration of attenuator settings below 100% (or of other repetition rates) against the delivered average power is available, and no pulse duration was measured for the 343 nm output. The available beam and pulse characterization refers to the laser-head output (Section 3.2). Pulse energy on target, fluence and delivered average power therefore cannot be reconstructed reliably from the available dataset, and only the recorded attenuator settings are reported. All processing was performed with circular polarization (Section 3.2); polarization was not varied as an experimental parameter, so polarization-dependent effects cannot be assessed from the present dataset.
(ix) Wavelength is confounded with repetition rate and nominal power setting in this dataset. The ultraviolet conditions were recorded at 4–50 kHz and 15–50% nominal power setting, and the infrared conditions at 200 kHz and 5% (Table 1); no condition is common to the two series. Combined with (viii), this means the available dataset cannot place the ultraviolet and infrared conditions on a common fluence or dose axis, and cannot attribute the difference in electrical outcome to wavelength alone. The laser-written sample set from the screening matrix is no longer available, so these limitations cannot be resolved retrospectively; they define the measurement program for a future sample campaign: measured spot size, delivered average power and fluence; Raman and XPS; higher-resolution and larger-area imaging; cross-sectional profiling; and replicate electrical measurements.
(x) An ablation threshold was not determined during the screening matrix. Because the focused spot size and delivered pulse energy are not recoverable from the available records, a quantitative threshold cannot be reconstructed retrospectively; determining it would require a dedicated fluence-resolved damage-threshold measurement on a new sample set.
(xi) More advanced thermal-transport modeling, including non-classical or fractional formulations, may also be useful in future work for describing transient heat transport in laser-converted carbonaceous structures [54], but such modeling is outside the scope of the present experimental dataset.

## 3. Materials and Methods

### 3.1. Materials

Commercial Kapton polyimide films (grade PV9100, 50 µm thick) were obtained from DuPont (Wilmington, DE, USA). They were cut into 1 cm × 1 cm squares, washed with acetone, isopropanol and deionized water and dried in air before laser processing.

The optical absorption of the Kapton PV9100 film was not measured in this work, and no transmission or absorption spectrum of the material used is available. No absorption coefficient is therefore reported for the present film at either processing wavelength. Published optical measurements on Kapton indicate strong attenuation in the near-ultraviolet [48], whereas femtosecond processing of polyimide at 1030 nm has been associated with nonlinear absorption pathways [39]. These literature observations provide qualitative context only and are not measurements of the present PV9100 films.

### 3.2. Laser System and Conductive Carbon Fabrication

All processing was performed with a single femtosecond laser source (Tangerine, Amplitude Systèmes, Pessac, France) integrated into a laser micromachining system by Oxford Lasers (Didcot, UK). The source emits at a fundamental wavelength of 1030 nm and provides frequency-doubled and frequency-tripled outputs; the fundamental (1030 nm; infrared, IR) and the third harmonic (343 nm; ultraviolet, UV) were used in this work. Each wavelength is delivered to the sample through its own optical path, which comprises a beam expander(Didcot, UK), a motorized attenuation system (a half-wave plate and an analyzer, adjustable from 0 to 100%, a quarter-wave plate, galvanometric scanning mirrors(Didcot, UK), and an F-theta focusing lens(Sill Optics GmbH, Wendelstein, Germany); these components were supplied as part of the Oxford Lasers system. The IR path used a Sill Optics S4LFT4010-328 F-theta lens (Sill Optics GmbH, Wendelstein, Germany; nominal focal length 100 mm; manufacturer design wavelength 1064 nm, specified wavelength range 1030–1090 nm), and the UV path used a Sill Optics S4LFT4110/075 F-theta lens (Sill Optics GmbH, Wendelstein, Germany; nominal focal length 109.4 mm; specified by the manufacturer for 355 nm). The beam, measured after the beam expander and before the focusing lens, had a Gaussian intensity distribution with a full 1/e^2^ beam diameter of 11.5 mm in the IR path and 4.6 mm in the UV path. These beam diameters were measured after the processing matrix was completed, with the same optical configuration as that used for the experiments; the magnification of each beam expander was not documented. The quarter-wave plate converts the beam to circular polarization, so all processing reported here was performed with circularly polarized light. The nominal power setting (%) reported in Table 1 and Table 2 is the setting of the motorized attenuator; it is not a measured average power at the sample (Section 3.2.1 and Section 3.2.2).

The laser-induced conductive carbon was produced by direct laser writing on the Kapton film surface. The films were mounted on a flat sample holder and flattened as far as possible, since focusing on the Kapton surface is critical to the resulting layer properties. The square 1 cm × 1 cm fields were filled in a single pass by a linear raster of parallel scan lines written along the X direction, generated from the computer-aided design (CAD) pattern imported into the scanning system, with a center-to-center line (hatch) spacing of 6.7 µm. As specified in the machining (G-code) program, successive lines were written in alternating directions (bidirectional scanning). All writing was performed in ambient air at room temperature. From the recorded scan speed v and repetition rate f, the pulse-to-pulse spacing along each scan line, Δx = v/f, was 0.10–0.25 µm for the IR conditions and 0.30–1.0 µm for the UV conditions (Table 1). The overlap between successive pulses and between adjacent scan lines depends on the focused spot diameter at the sample, which was not measured (Section 3.2.2); it is therefore not quantified here.

Characterization data supplied with the laser source, measured at the laser-head output at a repetition rate of 200 kHz, give measured center wavelengths of 1031 nm for the fundamental and 344.4 nm for the third harmonic, and second-moment (4σ) beam diameters of 2.19 mm × 2.05 mm at 1030 nm and 1.47 mm × 1.36 mm at 343 nm. At 1030 nm, the beam-quality factor is M^2^ = 1.26 and 1.25 along the two principal axes. The measured transverse line profiles at both wavelengths closely follow Gaussian fits, so the spatial intensity distribution at the laser-head output is near-Gaussian rather than flat-top; a near-Gaussian distribution is therefore also expected in the focal plane, although the focal-plane profile was not measured. The pulse duration was obtained from an autocorrelation measurement: assuming a sech^2^ temporal profile, I(t) ∝ sech^2^(1.763t/τ), the measured autocorrelation width of 0.474 ps (full width at half maximum, FWHM) corresponds to a pulse duration τ ≈ 308 fs (FWHM) for the fundamental (1030 nm) output. The autocorrelation trace shows weak shoulders at approximately ±0.6 ps delay, below approximately 20% of the peak, that are not reproduced by the sech^2^ fit. Because an autocorrelation is symmetric in delay, these shoulders cannot be assigned to pre- or post-pulses, and satellite pulses outside the 5 ps measurement window were not assessed. No pulse-duration measurement is available for the 343 nm output. All of these values refer to the pulses and beam at the laser-head output and were obtained before the processing matrix was written; no temporal characterization was performed further along the optical path, and the spatial beam parameters were not measured at the sample plane.

#### 3.2.1. UV Processing Configuration

The UV series used the third-harmonic output (343 nm). No measured pulse duration is available for this output (Section 3.2). The average power at the 100% attenuator setting and a repetition rate of 200 kHz (the 100% reference power) was 2.6 W, measured with a power meter mounted on the stage at the same level as the samples, directly beneath the focusing lens. In the UV configuration, the repetition rate could be set up to 200 kHz. The beam was delivered onto the Kapton surface through the UV optical path described in Section 3.2. For the UV parametric study, the repetition rate (4–50 kHz), nominal power setting (15–50%) and scan speed (2–40 mm s^−1^) were varied as indicated in Table 1. For each test, the 100% reference power was measured to monitor day-to-day variations in laser output. The attenuator percentage reported in Table 1 is the final setting used for that experiment. Because no calibration relating attenuator settings below 100% to the delivered average power is available, these settings are reported as nominal values and are not converted to watts. Accordingly, the delivered power cannot be assumed to scale linearly with the nominal attenuator percentage. The UV conditions were run at repetition rates below the 200 kHz at which the reference power was measured; therefore, no delivered average power is assigned to those processing conditions.

#### 3.2.2. IR Processing Configuration

The IR series used the fundamental output of the same source (1030 nm). The 100% reference power at 200 kHz, measured in the same way at the sample level (Section 3.2.1), was 13.6 W. In the IR configuration, the repetition rate could be programmed up to 800 kHz. The IR-O series was processed at 200 kHz using a nominal power setting of 5% and scan speeds of 20, 30, 40 and 50 mm s^−1^. As for the UV series, the 100% reference power was measured for each test to monitor laser-output variations, the percentage setting was adjusted when necessary, and the 5% setting reported in Table 1 is the final nominal attenuator setting used. No calibration relating settings below 100% to the average power delivered to the sample is available. The focused spot diameter at the sample was not measured for either wavelength: the processing system has no beam-profiling camera at the focal plane, and no ablation-crater measurement from which the spot diameter could be deduced was made. For reference only, the manufacturer datasheet of the IR lens lists a nominal 1/e^2^ focus diameter of 19.45 µm, together with an input beam diameter of 10 mm (1/e^2^) and a design wavelength of 1064 nm; this is a manufacturer specification for those conditions, which differ from the present ones (1030 nm, 11.5 mm), and not a measured spot size. The UV lens datasheet gives no focus diameter. Accordingly, delivered average power, pulse energy on target and fluence are not reconstructed here, and the original attenuator settings are reported.

The focal position was determined empirically by producing test marks while varying the Z-stage position in 50 µm increments and repeating the procedure over several laser-power levels, decreasing step by step. The resulting marks were inspected using the system microscope/camera, and the optimal focal position was identified from the region producing the smallest and most sharply defined marks. This procedure was used to locate the best-focus region without directly measuring the optical focal-spot diameter. According to the laser operator, the appearance of the marks typically changes little over a Z range of approximately 100–200 µm around best focus, which the operator attributes to the low numerical aperture of the focusing lenses; this range depends on the material and was not quantified for the present Kapton films. All laser-treated conditions were re-coded in terms of wavelength and experiment (Table 1), where UV-L denotes low-dose UV screening, UV-H denotes higher-dose UV screening, and IR-O denotes IR optimization. Here, the labels “low-dose” and “higher-dose” refer only to the relative nominal processing settings used in the screening matrix; the delivered optical dose cannot be reconstructed from the available records.

### 3.3. Optical Documentation

Macroscopic photographs of the processed arrays were acquired using a Leica S9 i stereomicroscope (Leica Microsystems GmbH, Wetzlar, Germany) equipped with an integrated 10 MP digital camera under ambient laboratory illumination at a working distance of 120 mm. Because image acquisition was not standardized for reflectance or illumination, the photographs are used exclusively for qualitative visual comparison, and no quantitative optical metrics were derived from them.

### 3.4. Structural and Morphological Characterization

Surface morphology was examined by field-emission scanning electron microscopy (SEM; Supra 55VP, Carl Zeiss Microscopy GmbH, Oberkochen, Germany) at IEMN, University of Lille. The infrared-series micrographs were acquired at 2.00 kV using an in-lens detector, with working distances of 3.7–4.6 mm. Laser-written samples were mounted on aluminum stubs with conductive carbon tape providing the grounding path. No conductive over-coating was applied, and the laser-written regions showed little charging. SEM was employed as a screening tool across the laser-written arrays, and micrographs were available for the conditions in which a developed surface structure was observed. The micrographs were acquired at broader and finer length scales for each condition, but magnification and working distance were not standardized across the series. The micrographs are used in this work as morphological evidence only.

### 3.5. Electrical Characterization

The UV- and IR-processed samples were subjected to the same electrical characterization procedure. Sheet resistance was measured in the van der Pauw configuration using an HL5500-series Hall measurement system. Four small indium point contacts (estimated diameter < 0.5 mm) were manually positioned at the corners of each 1 cm × 1 cm square sample. These contacts remained fixed throughout testing and were not repositioned for spatial mapping. The HL5500 system automatically processed the standard van der Pauw electrical permutations to generate a single sheet resistance value per sample. This instrument-level value represents averaging over the individual configurations rather than spatial mapping or independent sample replication. Samples for which this procedure yielded no sheet-resistance value are reported as showing “no measurable response,” meaning that no electrical conduction was detected under the applied van der Pauw procedure. The instrument-specific detection limit cannot be reconstructed from the available records.

The van der Pauw method determines sheet resistance by combining electrical resistance measurements obtained using different current and voltage contact configurations, without requiring the conductive-layer thickness as an input. Accordingly, the electrical values are reported as sheet resistance, R_s_, in ohms per square (Ω/□). Van der Pauw–Hall configurations have also been applied to the electrical transport characterization of graphene materials [55]. The method assumes a homogeneous and isotropic conducting layer of uniform thickness, with sufficiently small electrical contacts positioned at the specimen boundary [56]. The laser-converted layer is porous, and its thickness uniformity was not verified. The reported values should therefore be treated as effective sheet resistances of the converted surface region. No conductive-layer thickness was measured, and consequently no bulk resistivity is reported.

Potential systematic contributions to the sheet-resistance uncertainty include the instrumental precision of the measurement configurations, finite contact size, and local directional variation arising from the laser raster orientation. Because each sample was characterized by a single measurement, these values are presented as descriptive nominal indicators across the individual processing conditions. The original individual readings and the underlying instrument records are no longer available. The systematic contributions listed above therefore cannot be quantified, and no measurement uncertainty is assigned to the reported values. Although the four-contact van der Pauw configuration minimizes the contributions of contact and lead resistances to the measured sheet resistance, two-terminal contact characterization of future samples will be required for device-level electrode qualification.

### 3.6. Quantitative Image Analysis

The SEM micrographs were quantified in Python 3.12 using Otsu thresholding [57] as implemented in scikit-image 0.26.0 [58], together with SciPy 1.17.1 [59] and NumPy 2.4.4; figures were produced with Matplotlib 3.10.8. For the IR-O analysis, calibration was taken from the original instrument databar of each recovered source micrograph. These source images are reproduced with their original databars intact in Figure 4. Absolute spatial calibration was derived from the instrument-generated 1 µm scale bars in the two 10.00 k× micrographs, measured by a sub-pixel half-maximum convention; the two independent determinations agree to better than 0.1%. The nominal 2:1 magnification ratio was used only to transfer that calibration consistently to the 20.00 k× acquisitions, which carry a disproportionately large edge-measurement uncertainty because of their short rendered 200 nm bars; nominal magnification was not used to establish absolute scale. The resulting field dimensions carry a calibration uncertainty of approximately ±2%. The apparent open-porosity area fraction is dimensionless and is unaffected by this spatial calibration uncertainty. The images were segmented by Otsu thresholding after median filtering and background flattening. From the segmented images, the apparent open-porosity area fraction was extracted. For the IR-O series, one micrograph was analyzed per condition, using the highest-magnification image available for that condition (Figure 4): panel (b), 20.00 k×, for IR-O1; panel (d), 10.00 k×, for IR-O2; panel (f), 20.00 k×, for IR-O3; and panel (h), 10.00 k×, for IR-O4. Further micrographs acquired at other magnifications are shown in Figure 4 but were not used for quantification.

To avoid bias from the differing acquisition magnifications, the four images were brought to a common analysis array of 351 × 238 pixels, corresponding to a physical field of approximately 5.4 × 3.7 µm, set by the smallest of the four source fields. The 20.00 k× images were decimated by exactly a factor of two using block averaging; the 10.00 k× images were left at their native sampling. Deterministic centered crops were then extracted from all four arrays. No image was upsampled at any stage. Processing was identical for all four conditions: a 3 × 3 disk median filter, background flattening by subtraction of a Gaussian-smoothed copy with a 25-pixel standard deviation, and an Otsu threshold computed once per condition on the whole common field. Pixels below the threshold were counted as open pore space. No morphological post-processing, object filtering, or border exclusion was applied.

Values are reported as mean ± standard deviation over six sub-fields, formed as a 3 × 2 tiling of the common field into blocks of 117 × 119 pixels that covers the analyzed array exhaustively. These sub-fields are spatial subsamples of one field of view rather than independent experimental replicates; the quoted dispersion therefore describes within-field spatial variability only and does not support inferential comparison between conditions. The sensitivity of the porosity estimate to the segmentation threshold was assessed by shifting the Otsu threshold by −10, −5, +5, and +10 intensity units on the 8-bit scale inherited from the source micrographs and recording the maximum absolute deviation of the resulting porosity. These values describe segmentation sensitivity and are not confidence intervals. No hypothesis test is applied to these descriptors. Quantitative morphology is restricted here to the apparent open-porosity area fraction. Pore-size, ligament-width and object-count descriptors are not reported because they could not be determined robustly from the available images. A resolution-sensitivity test showed that these descriptors depended on the sampling interval. Size-resolved morphological characterization therefore requires higher-resolution imaging (Section 2.7).

### 3.7. X-Ray Diffraction

X-ray diffraction (XRD) was performed on the infrared-processed sample with the lowest reported sheet resistance (IR-O1) using a Rigaku Ultima IV diffractometer (Rigaku Corporation, Tokyo, Japan) with Cu Kα radiation (λ = 1.5406 Å), operated at 40 kV and 40 mA, and 2θ scans were recorded over the range of 5–60°. An as-received Kapton film was also characterized as a reference using the same θ–2θ measurement geometry (Figure 5).

## 4. Conclusions

The results presented in this work indicate that the formation of conductive laser-converted carbon layers on Kapton by femtosecond processing depends on the combination of processing conditions investigated. In this dataset, the ultraviolet and infrared conditions differ in repetition rate and nominal power setting as well as in wavelength, so the contrast between them cannot be assigned to wavelength alone. The UV laser-processing conditions studied resulted in visible darkening in most cases but no measurable electrical response under the applied van der Pauw procedure. The only ultraviolet condition for which micrographs are available, UV-L4, showed a discontinuous porous morphology. Within the ultraviolet parameter space explored in the screening matrix, no evidence of a continuous electrically conductive network was obtained.

In contrast, IR femtosecond laser treatment led to electrically conductive porous carbon layers throughout the investigated optimization series. The reported sheet resistance increased from 65.1 Ω/□ at 20 mm s^−1^ to 95.7 Ω/□ at 40 mm s^−1^ and was 93.3 Ω/□ at 50 mm s^−1^, at a fixed repetition rate and nominal power setting. Because no measurement uncertainty could be assigned to the reported values (Section 3.5), the differences are descriptive rather than statistically established, and the statistical distinguishability of the values at 40 and 50 mm s^−1^ cannot be assessed using the available measurement information. However, image analysis of the optimization series did not resolve a difference in apparent open-porosity area fraction among the four conditions within the limits of one quantitatively analyzed field per condition. The observed electrical variation is therefore not accounted for by the apparent open-porosity area fraction measured from the available fields. Larger-scale properties of the converted layer, such as its continuity across adjacent scan lines (6.7 µm hatch spacing) and its conversion depth, remain plausible contributors but were not measured.

IR-O1 had the lowest reported sheet resistance (65.1 Ω/□) among the four investigated IR-O conditions. The additional broad reflection in the graphite (002) region observed by XRD, which probes the illuminated area, supports the presence of graphitic stacking in the IR-O1 layer; the electron micrographs describe the morphology of that layer but do not identify which imaged features are graphitic.

Overall, the principal contribution of this study is the identification, within a single femtosecond-processing matrix, of UV parameter sets that produced no measurable electrical response and four IR processing conditions that yielded measurable sheet resistance, together with an electrical and microstructural assessment of the latter. Beyond these contrasting electrical outcomes, the study shows that, under the tested conditions, visible darkening and local pore formation were not sufficient indicators of a measurable electrical response, that the measured apparent open-porosity area fraction alone did not resolve the origin of the observed sheet-resistance differences among the IR conditions, and that graphitic stacking is supported for IR-O1, the only laser-processed sample examined by XRD.

IR-O1 yielded the lowest reported sheet resistance, 65.1 Ω/□, while comparison with published values for laser-converted polyimide, which is indicative only (Section 2.6), suggests that further optimization would be required to approach the lowest values reported in the literature. Within these limitations, the study provides an experimental basis for further investigation and optimization of femtosecond-written conductive carbon on flexible Kapton substrates.

## Figures and Tables

**Table 1:** Coding system and processing parameters for femtosecond laser treatment of Kapton substrates. All fields were written in a single pass as a linear raster along the X direction with a hatch spacing of 6.7 µm (Section 3.2). Laser power is given as the nominal power setting, i.e., the setting (0–100%) of the motorized attenuator, as finally used in the screening matrix; the 100% reference powers at 200 kHz, measured at the sample level, were 13.6 W (IR) and 2.6 W (UV) (Section 3.2.1 and Section 3.2.2), but no calibration relating lower settings to the average power delivered to the sample is available, and the setting is therefore not converted to watts (Section 3.2). Δx = v/f is the pulse-to-pulse spacing along the scan line, calculated from the recorded scan speed v and repetition rate f.

Group	Code	Wavelength	Repetition Rate (kHz)	Nominal Power Setting (%)	Scan Speed (mm s^−1^)	Δx = v/f (µm)
UV-L	UV-L1	UV	4	15	2	0.50
UV-L	UV-L2	UV	4	15	4	1.00
UV-L	UV-L3	UV	50	15	15	0.30
UV-L	UV-L4	UV	50	15	20	0.40
UV-L	UV-L5	UV	50	15	30	0.60
UV-L	UV-L6	UV	50	15	40	0.80
UV-H	UV-H1	UV	4	50	2	0.50
UV-H	UV-H2	UV	4	50	4	1.00
UV-H	UV-H3	UV	25	30	15	0.60
UV-H	UV-H4	UV	25	30	20	0.80
UV-H	UV-H5	UV	50	30	20	0.40
UV-H	UV-H6	UV	50	30	30	0.60
IR-O	IR-O1	IR	200	5	20	0.10
IR-O	IR-O2	IR	200	5	30	0.15
IR-O	IR-O3	IR	200	5	40	0.20
IR-O	IR-O4	IR	200	5	50	0.25

**Table 2:** Electrical screening summary.

Group	Code	λ	f (kHz)	P_nom_ (%)	v (mm s^−1^)	R_s_ (Ω/□)
UV-L	UV-L1–UV-L6	UV	4–50	15	2–40	no measurable response
UV-H	UV-H1–UV-H6	UV	4–50	30–50	2–30	no measurable response
IR-O	IR-O1	IR	200	5	20	65.1
IR-O	IR-O2	IR	200	5	30	76.7
IR-O	IR-O3	IR	200	5	40	95.7
IR-O	IR-O4	IR	200	5	50	93.3

λ = wavelength; f = repetition rate; P_nom_ = nominal power setting (motorized attenuator setting, 0–100%); v = scan speed (Section 3.2). “No measurable response” denotes a screening-level negative outcome (Section 3.5). One van der Pauw sheet-resistance value is reported per IR-O condition, based on four-contact measurements with fixed indium contact positions. The original individual readings are unavailable, and no measurement uncertainty is assigned (Section 3.5).

**Table 3:** Apparent open-porosity area fraction and sheet resistance of the IR-O series.

Code	v (mm s^−1^)	Apparent Open-Porosity Area Fraction (%)	R_s_ (Ω/□)
IR-O1	20	58.1 ± 1.2	65.1
IR-O2	30	59.9 ± 3.3	76.7
IR-O3	40	53.4 ± 4.8	95.7
IR-O4	50	55.2 ± 3.3	93.3

v = scan speed; R_s_ = sheet resistance (Ω/□); one van der Pauw sheet-resistance value is reported per condition without a quantified measurement uncertainty (Section 3.5). The apparent open-porosity area fraction is reported as mean ± standard deviation calculated over six spatial sub-fields of one field of approximately 5.4 × 3.7 µm (Section 3.6); the dispersion describes within-field spatial variability only. The maximum absolute deviation over the threshold sweep was 17.6, 17.8, 7.3, and 12.8 percentage points for IR-O1, IR-O2, IR-O3, and IR-O4, respectively, and represents segmentation sensitivity, not confidence intervals.

## Data Availability

The processed data supporting the findings of this study are available from the corresponding authors upon reasonable request. Some original raw data from the experimental screening matrix, including the underlying sheet-resistance measurement records, are no longer retrievable. The laser-written sample set is also no longer available for additional retrospective characterization.
